# Higher Utilization of Social Services Is Associated with Higher Language Scores in Children from Deeply Impoverished Urban Families

**DOI:** 10.3390/ijerph17228607

**Published:** 2020-11-19

**Authors:** Morgan A. Finkel, Sonya V. Troller-Renfree, Kimberly G. Noble

**Affiliations:** 1 Department of Pediatrics, Columbia University Irving Medical Center, 622 West 168th St., VC 417, New York, NY 10032, USA; maf2260@cumc.columbia.edu; 2 Department of Biobehavioral Sciences, Teachers College, Columbia University, Box 199, 525 West 120th St., New York, NY 10027, USA; svt2110@tc.columbia.edu

**Keywords:** socioeconomic disparities, child development, social services, language development, infant development

## Abstract

Language development has been consistently linked with socioeconomic status (SES), with children from lower SES backgrounds at higher risk for language delays. The objective of this study is to investigate the relationship between familial social service use and language development during the first year of life. Thirty-one low-income mothers and their infants were recruited from the New York metropolitan area. Mothers provided information about demographics and utilization of social services (Women, Infants, and Children (WIC), food stamps, Medicaid, and public housing). Infant language skills were assessed using the Preschool Language Scale. Multiple linear regressions were used to investigate the relationship between social service use and language skills. We found that the number of social services utilized was not an overall significant linear predictor of language skills. However, social service use interacted with poverty level to predict language skills. Specifically, for families living in deep poverty, higher service use significantly predicted higher infant language scores (β = 3.4, *p* = 0.005). These results suggest that social services may be an appropriate target to help narrow socioeconomic disparities in language development.

## 1. Introduction

Extensive research suggests that family socioeconomic factors, such as parental educational attainment and family income, are associated with children’s educational achievement, health, and well-being [1,2]. Language development, in particular, has been consistently linked with socioeconomic status (SES), with children from lower SES backgrounds at higher risk for language delays [3,4]. Health care providers working in underserved areas may encounter patients with severe language delays at four times the national average [5]. As language development has been consistently linked to literacy and later school achievement, these disparities are of particular interest to pediatricians, public health officials, and anyone concerned about the long-term success of children [6,7,8,9].

As of 2017, 8% of American children were living in deep poverty or residing in homes at or below 50% of the Federal Poverty Level [10]. Children living in deep or extreme poverty face severe material deprivation and are more commonly exposed to other poverty-related risks, such as residential crowding [11]. Children in deep poverty are also more likely than those living in less extreme poverty to experience family adversities such as poor parental physical health and mental health, parental stress, and poor social support, all of which have been linked to developmental risk in children [12]. Prior research finds that the direct relationship between family income and later school completion is largest in the lowest income groups [13]. Researchers and policymakers alike have recommended programs that target deep and persistent poverty in early childhood [12,14].

Social services, including the Supplemental Nutrition Assistance Program (SNAP), the Special Supplemental Nutrition Program for Women, Infants and Children (WIC), Medicaid, and housing assistance, prevent many families from falling into deep poverty [15]. These services provide financial support to at-risk families and improve outcomes in early childhood [16]. By helping to provide basic nutrition, health care, and housing, these services directly affect the nourishment and health of children while reducing financial stress in the household. SNAP has been shown to partially buffer the effect of food insecurity on poor health ratings in children, while evidence suggests that enrollment in WIC helps to lessen the positive associations between familial stressors and overall poor child health and childhood obesity [17,18]. Enrollment in WIC and SNAP is also associated with decreased reports of child abuse and neglect, and decreased rates of nutritional related health problems such as anemia and failure to thrive [19]. Increased Medicaid eligibility and utilization have also been shown to improve pediatric health measures, including child mortality [20,21]. Families who access subsidized housing are better able to access medical care and less likely to have children who are underweight, as compared to eligible families who did not access housing assistance [22,23].

Less is known about how the receipt of social services relates to measures of early child development. Social services such as WIC and Medicaid decrease the overall economic stress of the home while also freeing up both money and time for parents. These benefits together may create a home environment more conducive to learning for young children. For these reasons, social service utilization may be an important and available tool to close socioeconomically-driven disparities in early childhood development.

Research has found that children from families with food insecurity are at higher risk for developmental delays, and one study found that children receiving WIC benefits had better developmental outcomes than their matched peers who did not receive WIC [24,25,26]. A recent study found similar results related to SNAP. Families who utilized SNAP had infants and toddlers with lower odds of being at developmental risk, as compared to likely eligible families who did not utilize the program [27]. SNAP utilization has also been connected to improved academic achievement in school age children [16,28]. Crowding at home has been associated with worse developmental outcomes, and residential mobility has been negatively associated with school achievement [29,30]. Studies assessing the relationship between public housing and child development are few, with one large study finding inconsistently significant effects of public housing on a measure of toddler vocabulary [31]. In contrast, a study assessing the relationship between public housing and academic achievement in older children found a beneficial effect of public housing on educational outcomes after controlling for possible confounders [32]. Public health insurance expansion during early childhood has been linked to improved reading scores when children reach elementary school and higher high school graduation rates [33,34].

Many families in New York City (NYC) rely on the social services provided by the government for financial support. As of 2019, 1.6 million New Yorkers (nearly 20% of the city population) utilize SNAP, and over 200,000 pregnant women and children under 5 receive benefits from WIC [35,36]. Over 4.4 million low income New Yorkers depend on Medicaid for health insurance coverage, and about 564,000 New Yorkers live in public housing or receive housing assistance [37,38]. One in 15 New Yorkers lives in public housing.

Little is known about whether the number of these social services utilized by families impacts child development and whether any such associations can be seen as early as infancy. This paper seeks to address the following question: Is accessing a higher number of social services associated with improved developmental outcomes for young children living in poverty? More specifically, we investigate whether familial utilization of WIC, SNAP, Medicaid, and housing assistance are associated with children’s language scores during their first year of life, and whether the relation between social service utilization and language scores varies as a function of the depth of poverty. We hypothesize that families who are most in need economically (i.e., those living in deep poverty) will have children whose language skills benefit the most from use of services. At a time when familial access to social services is being threatened by actions such as public charge and other funding cuts, it is important to understand their impacts on child development.

## 2. Materials and Methods

Participants constituted a subset of a larger cross-sectional study examining the association between early experiences and infants’ language, memory, and brain development. In the larger study, a cohort of 94 socioeconomically diverse mother–infant pairs were recruited from the New York metropolitan area and invited to participate when the infants were 6, 9, or 12 months of age (32 six-month-old, 31 nine-month-old, 31 twelve-months-old). The convenience sample was recruited via flyers in the community surrounding Columbia University in upper Manhattan, at a local WIC clinic, community events, and the laboratory website. The neighborhood surrounding the university is densely urban and diverse in terms of race, ethnicity, and socioeconomic status. Inclusionary criteria for children were as follows: (1) between 5.5 and 12.5 months of age, (2) born at or after 36 weeks of gestation, and (3) without neurological or developmental complications.

Mothers completed computerized questionnaires to provide demographic information, including familial income, familial size, and years of parental education. An income-to-needs (ITN) ratio was calculated for each child’s family by dividing the total combined familial annual income by the national federal poverty level for a family of that size, for the specified year or the most recent year available. The present study is limited to the 31 families with an ITN of 1.3 or less, rendering them eligible for all four of the following New York social services at the time of assessment: SNAP, WIC, housing assistance, and parental Medicaid. In this subsample, fifteen of the infants were 12 months old, eleven infants were 9 months old, and five infants were 6 months old. A majority of mothers identified their children as Latinx (61%) (Table 1). Mothers were asked to indicate whether or not their family was currently utilizing each of the social services listed above at the time of assessment.

Research staff assessed infant language abilities using the Preschool Language Scale 5^®^ (PLS) in English or Spanish, depending on the primary language reported by the family. The PLS is an interactive assessment of developmental language skills that has been validated for use in children from birth through 7 years [39]. It includes an Auditory Comprehension Score (ACS) and an Expressive Communication Score (ECS) that are summed to get a Total Standard Score (TSS). Sub-scores and total scores are standardized for age with a mean score of 100 and a standard deviation of 15.

Linear regressions were conducted to assess the relation between the number of social services utilized (0–4) and PLS total and subscale scores. Covariates included average parental education in years, familial ITN, and age of child at assessment. We additionally examined whether family ITN statistically moderated the association between social service utilization and language scores, followed by stratified analyses when appropriate. SPSS (IBM, Version 25, Armonk, New York, NY, USA) was used for all analyses.

All subjects gave their informed written consent for inclusion in their primary language before they participated in the study. The study was conducted in accordance with the Declaration of Helsinki, and the protocol was approved by the Teachers College, Columbia University IRB (Protocol Number 15-436).

## 3. Results

On average, families utilized 2.2 (SD = 1.2) of the four services for which they were eligible, with SNAP being the most highly utilized service (77%) and housing assistance the least accessed service (26%) (Table 1). Overall, these families were living at the extreme end of the income spectrum with an average ITN of 0.62 (SD = 0.4). The average parental educational attainment was 12.1 years (SD = 3 years). Average PLS scores were slightly below average (Total Standard Score: 96.3 ± 10.8, Auditory Comprehension Score: 98.7 ± 10.4, Expressive Comprehension Score: 94.6 ± 14.0). Child age at assessment and mean parental education were significantly associated with Total Standard Scores and were therefore included in all models (Table 2). Older age at assessment was inversely related to total standard score (β = −2.3, *p* = 0.007), and average parental education was positively related to total standard score (β = 1.3, *p* = 0.04).

The number of social services utilized was not linearly related to language skills. However, social service use interacted significantly with family ITN to predict Expressive Communication Standard Scores (ITN × social service utilization: β = −14.1, *p* = 0.02) and approached significance for Total Standard Scores (ITN × social service utilization: β = −8.9, *p* = 0.05), suggesting that the depth of poverty moderated the relationship between social services utilization and language skill (Table 2).

**Figure 1:**
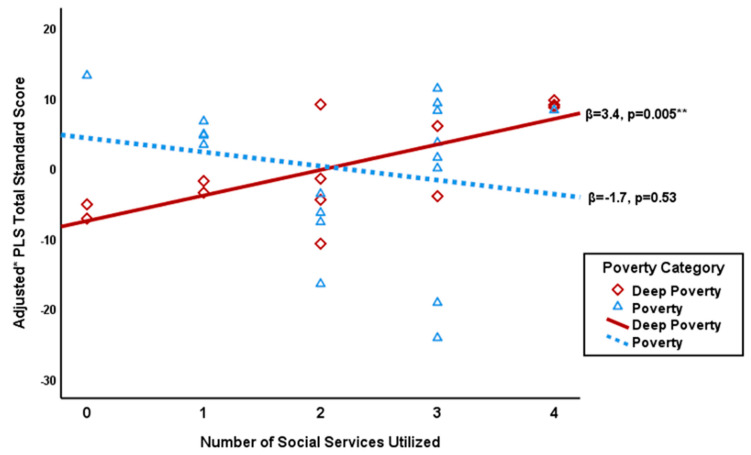
Social services utilized vs. adjusted* PLS total score stratified by poverty category. *Adjusted for average parental education and child age ** *p* < 0.01.

Probing this interaction with a stratified analysis, we found that for families living in deep poverty (ITN ≤ 0.5), the number of services utilized was significantly and positively related to Total Standard Scores (β = 3.4, *p* = 0.005) (Figure 1, Table 2). Further, for families living in deep poverty, the number of services utilized was borderline associated with both Auditory Comprehension Scores (β = 3.9, *p* = 0.05) and Expressive Communication Scores (β = 2.7, *p* = 0.09) (Table 2). There was no significant association between social service use and language outcomes among families with ITN greater than 0.5.

## 4. Discussion

For infants from families living in deep poverty, we found that an increased use of social services was associated with better language skills in the first year of life. To our knowledge, this is the first time this relationship has been investigated. Although the sample size is small and cross-sectional and should be replicated in larger longitudinal studies, it does highlight the potential positive relationship between the quantity of social services utilized and developmental outcomes. These results suggest that social services may be an effective way to alleviate socioeconomic disparities in development, specifically for children living in the most extreme poverty. These results are consistent with previous studies that have documented the positive effects of individual social services on child developmental and academic outcomes [24,27,28,32,33]. Most studies investigating the relationship between familial SES and child development focus on parenting behaviors of low SES parents, which can place the burden of change on low SES parents, but this study suggests that for families living in deep poverty, policies that reinforce the social safety net and increase financial supports for low income families may be a more appropriate target.

We found this link between the number of social services used and language development in a small sample of children in the first year of life. If replicated, we predict that this association would likely be more prominent in a sample of older children, whose language skill levels span a broader range [40]. Given that we were able to find this relationship in such young children suggests that interventions in the first months of life may have an impact on developmental outcomes of children. As described by Conti and Heckman, it is economically sensible to invest in preventing rather than “treating” disparities once they are already large [41].

There are many possible mechanisms that could explain the findings of this study. Utilization of social services decreases the overall financial stress of the home and may allow parents to focus their efforts on the needs of their children. This may lead to more time with their children and the creation of a richer home language environment, which has been associated with improved language scores in children [42,43,44]. It may also free up funds for educational resources such as books and games that are associated with cognitive development [45]. Improved nutrition from WIC and SNAP may support healthy brain development, which is a hypothesis supported by the results of a study that used large, nationally representative surveys to find a positive association between prenatal and early childhood exposures to WIC and cognitive outcomes in children [24]. Housing assistance may decrease housing instability and improve crowding, which has been connected to developmental risk [30,46]. Alternatively, it is possible that parents who can navigate the complexities of the system required to access services are more organized in general and better able to accommodate the developmental needs of their children. The mechanisms that explain this relationship need to be investigated further, using longitudinal or experimental research to attempt to disentangle directionality.

In addition to the primary outcomes investigated, we found that the percentage of services utilized by many families was low, with many families utilizing zero to two services out of the four services for which they were eligible. Not surprisingly, housing assistance, which often has limited availability with long wait lists, was the least utilized service. However, it was relatively surprising that other services, which are more easily accessed, were underutilized. Indeed, only 65% of eligible families were receiving supplemental food from WIC, and only 52% of eligible families were receiving parental Medicaid. Although this small sample is not intended to be representative, it does suggest that there may be significant opportunity for improvement in the utilization of services. More research is necessary to further understand barriers to enrollment and to develop programs to target improved enrollment for families with young children.

## 5. Conclusions

For infants from families living in deep poverty in an urban setting, the familial utilization of more social services was significantly associated with improved language scores in late infancy. The results of this study provide preliminary data to support the idea that social services are an appropriate target to help narrow socioeconomic disparities in infant language development. Further work is needed to examine whether these results can be generalized to other contexts. Additionally, longitudinal studies and trials that intervene to provide services and measure developmental outcomes are required to further understand this potential relationship as well as underlying mechanisms.

## Figures and Tables

**Table 1:** Child and parent characteristics and use of social services.

Child Characteristics		Count (% of Sample), n = 31
Age	6 months	5 (16)
	9 months	11 (36)
	12 months	15 (48)
Sex	Male	21 (68)
	Female	10 (32)
Race/Ethnicity	Black, Non-Hispanic	7 (23)
	Other, Non-Hispanic	1 (3)
	Hispanic of all races	19 (61)
	Prefer not to answer	4 (13)
**Descriptive Statistics**		**Mean ± s.d.**
Familial Income-to-Needs Ratio		0.62 ± 0.4
Mean Parental Education (years)		12.1 ± 3.0
Parental Age (years)		29.9 ± 6.5
Number of Services Utilized		2.2 ± 1.2
Language Scores:	Auditory Comprehension Standard Score	98.7 ± 10.4
	Expressive Communication Standard Score	94.6 ± 14.0
	Total Standard Score	96.3 ± 10.8
**Specific Service Utilization**		**Fraction Utilizing (% of Sample)**
WIC		20/31 (65)
SNAP		24/31 (77)
Housing Assistance		8/31 (26)
Parental Medicaid		16/31 (52)

**Table 2:** Multivariate linear regression modeling infant Preschool Language Scale 5^®^ (PLS) scores.

Variables	Total Standard Scores		Expressive Communication		Auditory Comprehension	
	β	*p*	β	*p*	β	*p*
**Full Sample**						
Mean Parental Education (yr.)	1.3	0.04 *	2.1	0.008 **	0.2	0.71
Age (mo.)	−2.3	0.007 **	−3.0	0.005 **	−1.2	0.18
ITN	−0.3	0.95	−5.9	0.35	5.2	0.36
Social Service Utilization	0.6	0.67	−0.7	0.71	2.0	0.22
ITN × Social Service Utilization	−8.9	0.05 ^	−14.1	0.02 *	−2.8	0.57
**ITN ≤ 0.5**						
Mean Parental Education (yr.)	1.4	0.004 **	1.6	0.01 *	1.0	0.14
Age (mo.)	−3.7	0.000 **	−3.8	0.002 **	−3.2	0.02 *
Social Service Utilization	3.4	0.005 **	2.7	0.09^	3.9	0.05 ^
**ITN > 0.5**						
Mean Parental Education	1.4	0.28	3.2	0.06	−0.7	0.57
Age	−1.3	0.34	−2.4	0.19	0.1	0.91
Social Service Utilization	−1.7	0.53	−3.3	0.37	0.3	0.91

^ *p* < 0.1, * *p* < 0.05, ** *p* < 0.01.

## References

[1] Bradley R.H., Corwyn R.F. (2002). Socioeconomic Status and Child Development. Annu. Rev. Psychol..

[2] Brooks-Gunn J., Duncan G.J. (1997). The Effects of Poverty on Children. Future Child..

[3] Fernald A., Marchman V.A., Weisleder A. (2013). SES differences in language processing skill and vocabulary are evident at 18 months. Dev. Sci..

[4] Rowe M.L. (2008). Child-directed speech: Relation to socioeconomic status, knowledge of child development and child vocabulary skill. J. Child. Lang..

[5] King T.M., Rosenberg L.A., Fuddy L., McFarlane E., Sia C., Duggan A.K. (2005). Prevalence and early identification of language delays among at-risk three year olds. J. Dev. Behav. Pediatr..

[6] Duff F., Reen G., Plunkett K., Nation K. (2015). Do Infant Vocabulary Skills Predict School-Age Language and Literacy Outcomes?. J. Child Psychol. Psychiatry.

[7] NICHD Early Child Care Research Network (2005). Pathways to Reading: The Role of Oral Language in the Transition to Reading. Dev. Psychol..

[8] (2010). Developing Early Literacy: Report of the National Early Literacy Panel.

[9] Walker D., Greenwood C., Hart B., Carta J. (1994). Prediction of School Outcomes Based on Early Language Production and Socioeconomic Factors. Child Dev..

[10] (2019). Children in Poverty.

[11] Roy A.L., Raver C.C. (2014). Are all risks equal? Early experiences of poverty-related risk and children’s functioning. J. Fam. Psychol..

[12] Ekono M., Jiang Y., Smith S. (2016). Young Children in Deep Poverty. Fact Sheet.

[13] Duncan G.J., Yeung W.J., Brooks-Gunn J., Smith J.R. (1998). How Much Does Childhood Poverty Affect the Life Chances of Children?. Am. Sociol. Rev..

[14] Duncan G.J., Brooks-Gunn J. (2000). Family Poverty, Welfare Reform, and Child Development. Child Dev..

[15] Fox L., Wimer C., Garfinkel I., Kaushal N., Nam J., Waldfogel J. (2015). Trends in Deep Poverty from 1968 to 2011: The Influence of Family Structure, Employment Patterns, and the Safety Net. RSF Russell Sage Found. J. Soc. Sci..

[16] Gassman-Pines A., Hill Z. (2013). How Social Safety Net Programs Affect Family Economic Well-Being, Family Functioning, and Children’s Development. Child Dev. Perspect..

[17] Black M.M., Quigg A.M., Cook J., Casey P.H., Cutts D.B., Chilton M., Meyers A., de Cuba S.E., Heeren T., Coleman S. (2012). WIC Participation and Attenuation of Stress-Related Child Health Risks of Household Food Insecurity and Caregiver Depressive Symptoms. Arch. Pediatr. Adolesc. Med..

[18] Cook J.T., Frank D.A., Berkowitz C., Black M.M., Casey P.H., Cutts D.B., Meyers A.F., Zaldivar N., Skalicky A., Levenson S. (2004). Food Insecurity Is Associated with Adverse Health Outcomes among Human Infants and Toddlers. J. Nutr..

[19] Lee B.J., Mackey-Bilaver L. (2007). Effects of WIC and Food Stamp Program participation on child outcomes. Child. Youth Serv. Rev..

[20] Aizer A. (2007). Public Health Insurance, Program Take-Up, and Child Health. Rev. Econ. Stat..

[21] Currie J., Gruber J. (1996). Health Insurance Eligibility, Utilization of Medical Care, and Child Health. Q. J. Econ..

[22] Meyers A., Cutts D., Frank D.A., Levenson S., Skalicky A., Heeren T., Cook J., Berkowitz C., Black M., Casey P. (2005). Subsidized housing and children’s nutritional status: Data from a multisite surveillance study. Arch. Pediatr. Adolesc. Med..

[23] Lee W.S., Beecroft E., Shroder M. (2005). The impacts of welfare reform on recipients of housing assistance. Hous. Policy Debate.

[24] Jackson M.I. (2015). Early childhood WIC participation, cognitive development and academic achievement. Soc. Sci. Med..

[25] Rose-Jacobs R., Black M.M., Casey P.H., Cook J.T., Cutts D.B., Chilton M., Heeren T., Levenson S.M., Meyers A.F., Frank D.A. (2008). Household Food Insecurity: Associations with At-Risk Infant and Toddler Development. Pediatrics.

[26] Drennen C.R., Coleman S.M., de Cuba S.E., Frank D.A., Chilton M., Cook J.T., Cutts D.B., Heeren T., Casey P.H., Black M.M. (2019). Food Insecurity, Health, and Development in Children Under Age Four Years. Pediatrics.

[27] Ettinger de Cuba S.A., Bovell-Ammon A.R., Cook J.T., Coleman S.M., Black M.M., Chilton M.M., Casey P.H., Cutts D.B., Heeren T.C., Sandel M.T. (2019). SNAP, Young Children’s Health, and Family Food Security and Healthcare Access. Am. J. Prev. Med..

[28] Frongillo E.A., Jyoti D.F., Jones S.J. (2006). Food Stamp Program Participation Is Associated with Better Academic Learning among School Children. J. Nutr..

[29] Leventhal T., Newman S. (2010). Housing and child development. Child. Youth Serv. Rev..

[30] Solari C.D., Mare R.D. (2012). Housing crowding effects on children’s wellbeing. Soc. Sci. Res..

[31] Fertig A.R., Reingold D.A. (2007). Public housing, health, and health behaviors: Is there a connection?. J. Policy Anal. Manage..

[32] Currie J., Yelowitz A. (2000). Are public housing projects good for kids?. J. Public Econ..

[33] Levine P.B., Schanzenbach D.W. (2009). The Impact of Children’s Public Health Insurance Expansions on Educational Outcomes. Forum Health Econ. Policy.

[34] Miller S., Wherry L.R. (2019). The Long-Term Effects of Early Life Medicaid Coverage. J. Hum. Resour..

[35] NYC Department of Social Services and NYC Mayor’s Office of Immigrant Affairs Fact Sheet: SNAP Enrollment Trends in New York City. https://www1.nyc.gov/assets/immigrants/downloads/pdf/Fact-Sheet-June-2019.pdf.

[36] NY State Department of Health, NYC Mayor’s Office of Immigrant Affairs and NYC Health Fact Sheet: WIC Enrollment Trends in New York City. https://www1.nyc.gov/assets/immigrants/downloads/pdf/fact-sheet-wic-enrollment-trends-february-2020.pdf.

[37] United Hospital Fund New York Counties by Population, Medicaid Enrollment, and Enrollment Rates (Table). https://uhfnyc.org/publications/publication/new-york-counties-by-population-medicaid-enrollment-and-enrollment-rates-table/.

[38] NYC Housing Authority NYCHA 2019 Fact Sheet. https://www1.nyc.gov/assets/nycha/downloads/pdf/NYCHA-Fact-Sheet_2019_08-01.pdf.

[39] Zimmerman I.L., Steiner V.G., Pond R.E. (2011). Preschool Language Scales.

[40] Noble K.G., Engelhardt L.E., Brito N.H., Mack L.J., Nail E.J., Angal J., Barr R., Fifer W.P., Elliott A.J. (2015). Socioeconomic Disparities in Neurocognitive Development in the First Two Years of Life. Dev. Psychobiol..

[41] Conti G., Heckman J.J. (2013). The Developmental Approach to Child and Adult Health. Pediatrics.

[42] Hart B., Risley T.R. (1995). Meaningful Differences in the Everyday Experience of Young American Children.

[43] Hoff E. (2003). The Specificity of Environmental Influence: Socioeconomic Status Affects Early Vocabulary Development Via Maternal Speech. Child Dev..

[44] Merz E.C., Maskus E.A., Melvin S.A., He X., Noble K.G. (2019). Socioeconomic Disparities in Language Input Are Associated with Children’s Language-Related Brain Structure and Reading Skills. Child Dev..

[45] Melvin S.A., Brito N.H., Mack L.J., Engelhardt L.E., Fifer W.P., Elliott A.J., Noble K.G. (2017). Home Environment, But Not Socioeconomic Status, is Linked to Differences in Early Phonetic Perception Ability. Infancy.

[46] Cutts D.B., Meyers A.F., Black M.M., Casey P.H., Chilton M., Cook J.T., Geppert J., Ettinger de Cuba S., Heeren T., Coleman S. (2011). US Housing insecurity and the health of very young children. Am. J. Public Health.

